# Phylogenetic analysis of the wild-type strains of canine distemper virus circulating in the United States

**DOI:** 10.1186/s12985-018-1027-2

**Published:** 2018-08-02

**Authors:** Eman Anis, Teresa K. Newell, Neil Dyer, Rebecca P. Wilkes

**Affiliations:** 1 Tifton Veterinary Diagnostic and Investigational Laboratory, College of Veterinary Medicine, University of Georgia, PO Box 1389, 43 Brighton Rd, Tifton, GA 31793 USA; 2 The Department of Virology, Faculty of Veterinary Medicine, University of Sadat, Sadat City, Egypt; 3 Veterinary Diagnostic Services Department, North Dakota State University, Dept. 7691, P.O. Box 6050, Fargo, North, Dakota 58105 USA

**Keywords:** Canine distemper virus, Phylogenetics, Targeted next generation sequencing, Lineages

## Abstract

**Background:**

Canine distemper (CD) is a highly contagious, systemic, viral disease of dogs seen worldwide. Despite intensive vaccination in developed countries, recent reports suggest both the re-emergence and increased activity of Canine distemper virus (CDV) worldwide, including the United States. CDV is an RNA virus of the genus Morbillivirus within the family *Paramyxoviridae*. Viral genomic RNA encodes six structural proteins. Of the six structural proteins, the hemagglutinin (H) gene has the greatest genetic variation and is therefore a suitable target for molecular epidemiological studies. The majority of neutralizing epitopes are found on the H protein, making this gene also important for evaluation of changes over time that may result in antigenic differences among strains. The aim of this study was to determine the phylogenetic relationship of CDV strains circulating in the US.

**Methods:**

Fifty-nine positive canine distemper virus samples collected from dogs from different regions and states from 2014 to 2017 were sequenced with a targeted next-generation sequencing (NGS) method. The sequences of the H, F, and P genes and the matrix-fusion (M-F) intergenic region of the amplified CDVs were analyzed individually.

**Results:**

Sequence analysis of the H gene revealed that there are at least 3 different lineages of CDV currently circulating in the US. These lineages include America-3 (Edomex), America-4, and a clade that was previously reported in association with an outbreak in Wyoming, which was linked to a domestic dog-breeding facility in Kansas in 2010. These lineages differ from the historically identified lineages in the US, including America-1, which contains the majority of the vaccine strains. Genetic differences may result in significant changes to the neutralizing epitopes that consequently may lead to vaccine failure. Phylogenetic analyses of the nucleotide sequences obtained in this study of the F and P genes and the M-F intergenic region with sequences from the GenBank database produced similar findings to the H gene analysis.

**Conclusions:**

The CDV lineages currently circulating in the US differ from the historically identified lineages America-1. Continuous surveillance is required for monitoring circulating CDV strains in the US, to prevent potential vaccine breakthrough events.

**Electronic supplementary material:**

The online version of this article (10.1186/s12985-018-1027-2) contains supplementary material, which is available to authorized users.

## Background

Canine distemper (CD) is a highly contagious, systemic viral disease of dogs seen worldwide. Canine distemper virus (CDV), the causative agent of CD, is an enveloped virus in the genus Morbillivirus of the family *Paramyxoviridae*. CDV has two types of glycoprotein spikes, hemagglutinin (H) and fusion (F) proteins, on the viral envelope [1]. Although neutralizing antibodies against both of these viral envelope glycoproteins have been detected, the antibodies to H protein are known to be crucial for protective immunity against CD. To date, the epitopes of CDV-H protein have not been mapped, but the H protein is known to have the greatest antigenic variation and is therefore a suitable target for molecular epidemiological studies [2, 3]. Epitopes on the H protein have been mapped for other morbilliviruses, including measles virus and rinderpest virus, and an immunodominant epitope has been described in a similar structural location for these two viruses, suggesting the overall antigenic structures of the H proteins in morbilliviruses are similar [4]. Therefore, this could potentially be extrapolated to CDV to evaluate for changes that may be associated with antigenic differences that have demonstrated between strain [5].

Based on phylogenetic analysis of the hemagglutinin (H) gene sequences and their variability, CDV is classified into genetic lineages distributed worldwide according to a geographic pattern [6]. The condition for lineage assignment states that two strains belong to the same lineage if they cluster together in the phylogenetic tree, and show an H amino acid divergence less than 4% [6]. At least 12 well-defined CDV lineages have been identified worldwide, denoted America-1 (vaccine strains), America-2, America-3, America-4, Europe 1/South America 1, Europe 2/Europe-wildlife, Europe 3/Arctic-like, Asia 1, Asia 2, South Africa, South America 2 and 3 [7–15].

Despite intensive vaccination in developed countries, recent reports suggest both the re-emergence and increased activity of CDV worldwide, including the US [8, 9, 14, 16, 17]. The circulating wild-type CDV detected from various parts of the world possess different hemagglutinin (H) gene and protein sequences from the currently available modified live vaccines [7]. This raises the question of whether the vaccines currently used efficiently protect against circulating field strains [5]. It is clinically important to determine the exact CDV strain/lineage circulating in the field and how it is genetically and antigenically related to the available vaccines. Therefore, the aim of this study was to investigate the apparent rise in CD clinical cases in dogs in the US and to determine the phylogenetic relationship of the circulating CDV strains to the currently used vaccines.

## Methods

### Samples and real-time RT-PCR

Fifty-nine positive CDV cases were kindly provided by veterinary diagnostic laboratories throughout the US (Kansas State University Veterinary Diagnostic Laboratory, North Dakota State University Veterinary Diagnostic Laboratory, University of Georgia Tifton Veterinary Diagnostic and Investigation Laboratory, University of Tennessee Veterinary Clinical Virology Laboratory, University of Illinois Veterinary Diagnostic laboratory, Mississippi State University Veterinary Research & Diagnostic Laboratory, Washington State University Animal Disease Laboratory, University of Clemson Veterinary Diagnostic Center, Iowa State University Veterinary Diagnostic Laboratory, and University of Wyoming State Veterinary Laboratory). All of the positive samples were obtained from clinically affected dogs. Fifty-seven out of the 59 samples were collected in the period from 2014 to 2017, and two samples were collected between 2004 and 2005. Samples in this study included multiple tissues (lung, brain, spleen, kidney and urinary bladder), bodily fluids (urine and EDTA whole blood), swabs (conjunctival and nasal swabs) and formalin fixed paraffin embedded tissue scrolls. Two of the labs submitted extracted nucleic acids for sequencing. Samples used for this study had been sent to each laboratory for diagnostic purposes and not all samples were submitted with complete histories. Therefore, we were not able to determine the vaccination history for most of the cases.

To confirm the detection of CDV from the submitted cases, real-time RT- PCR was performed. Viral nucleic acid was extracted using a commercial kit (DNeasy Blood and tissue kit, Qiagen, Valencia, CA) according to manufacturer’s instructions for each sample type. Tissues were pooled and macerated in a 1:1 volume of PBS and nucleic acids were extracted from the supernatant. Nucleic acids sent to the lab had been extracted with the MagMax Nucleic acid isolation kit (Thermo Fisher Scientific, Waltham, MA) or the QIAamp Viral RNA Mini Kit (Qiagen) according to the manufacturers’ protocols. All nucleic acids were stored at − 80 °C until tested. Real-time RT-PCR was performed as previously published [18] and Ct values of the samples ranged from 15 to 33.

### Genome sequencing using targeted next generation sequencing

Overlapping primers were designed and divided into two primer pools to amplify the complete CDV genome sequence (Additional file 1). Multiple primers per target were designed to amplify various CDV strains. These primers were designed using the Ion Ampliseq Designer (https://ampliseq.com/login/login.action), a primer design tool to create custom panels for targeted sequencing, and changes were made to the design with the assistance of the White Glove Team (Ion Torrent- Thermo Fisher Scientific, Waltham, MA).

RT-PCR and library preparation were performed on the Ion Chef using the Ampliseq™ kit for Chef DL8 (Thermo Fisher Scientific) according to the manufacturer’s protocol. This kit allowed the preparation of 8 barcoded ion Ampliseq™ libraries per Ion Chef run (8 different clinical cases). Then, 20-50pM of the 8 mixed libraries were templated and loaded on an Ion 314™ chip using the Ion Chef instrument with the Ion PGM™ kit (Thermo Fisher Scientific) according to the manufacturer’s instructions. Finally, the libraries were sequenced on an Ion Torrent Personal Genome Machine (PGM, Thermo Fisher Scientific) sequencer with the Ion PGM Hi-Q™ sequencing kit (Thermo Fisher Scientific) according to the manufacturer’s instructions.

### Data analysis

Ion Torrent machine supplied cloud-based bioinformatics programs were used initially to assemble and align the amplified sequence with a reference CDV genome. In this study CDV strain A75/17, GenBank accession number AF164967, was used as reference strain. Then the sequences of the H, F, and P genes and the matrix-fusion (M-F) intergenic region of the amplified CDVs were analyzed individually using Geneious software version 11.0.3 (www.geneious.com). Available complete or partial H, F and P genes as well as the M-F region representing the major available CDV lineages were downloaded from GenBank and each gene/region was aligned to the amplified CDV gene sequences using MAFFT [19]. Consensus phylogenetic trees were also generated for H, F, and P genes and the M-F region within Geneious software using the unweighted pair-group method with 1000 bootstrap replicates.

The H sequences from representative samples from each clade were translated and an alignment between amino acid sequences 364 to 392 was generated in Geneious using MUSCLE.

## Results

**Fig. 1:**
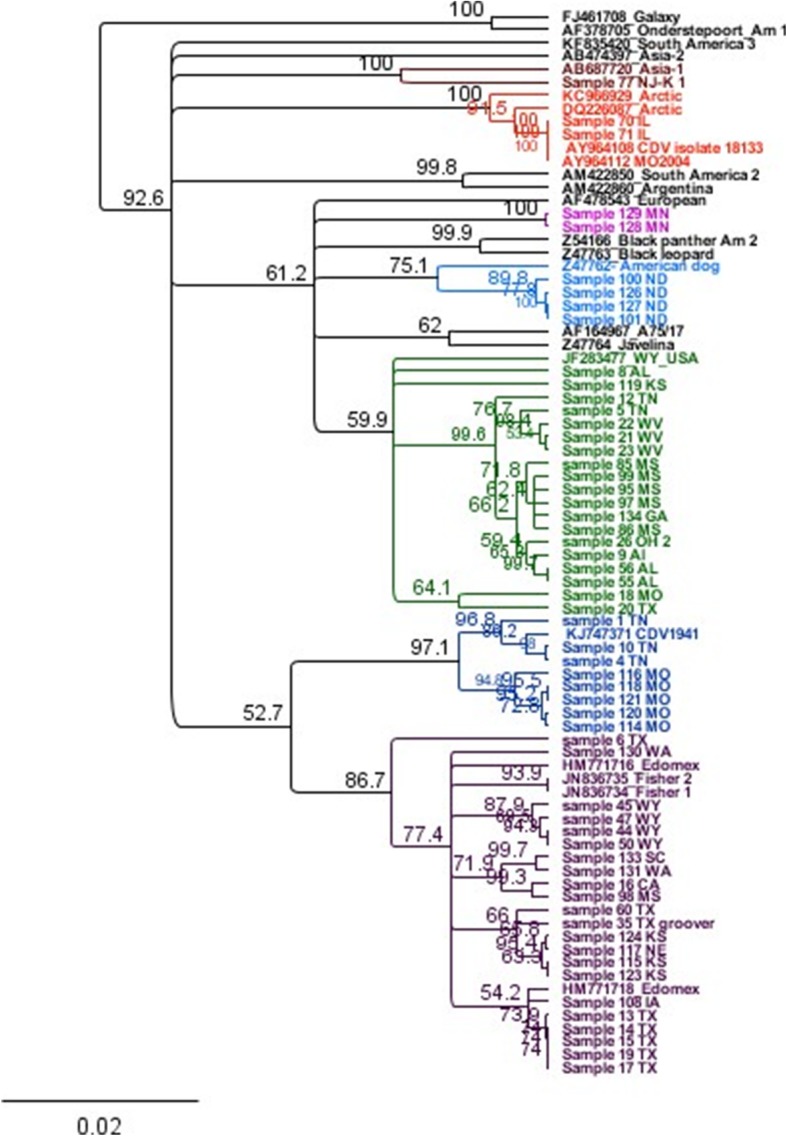
Phylogenetic tree for H gene showing strains of CDV circulating in various region of the US

**Fig. 2:**
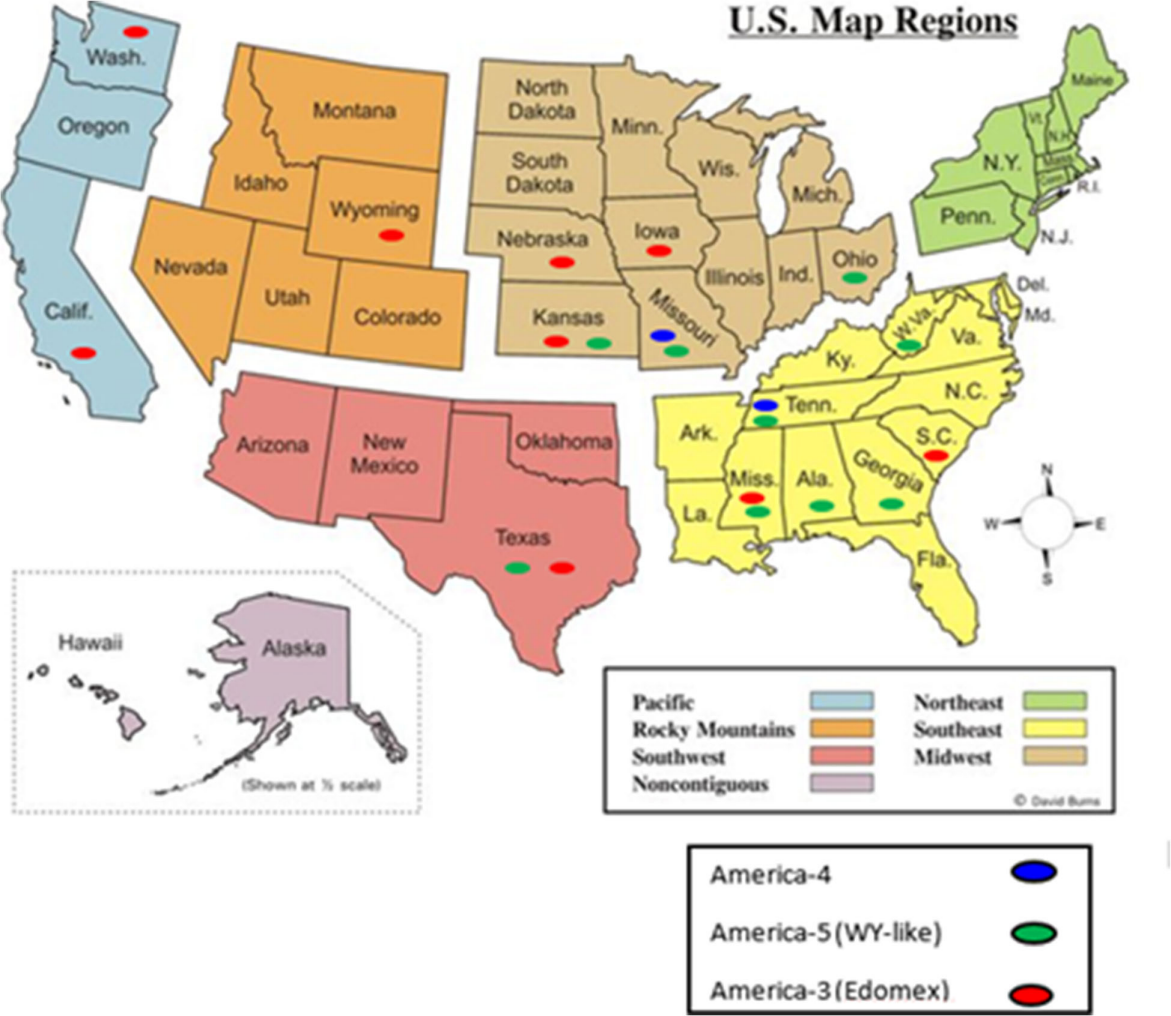
Map to show the distribution of the three main CDV lineages currently circulating in the US

The targeted NGS assay was able to amplify CDV strains collected from various geographical regions/states. Though the primers used in this assay were designed to amplify the complete CDV genome, there were gaps/ missing nucleotide sequences scattered in various regions of the amplified genome. Therefore, either partial or nearly complete sequence of the CDV H, F and P genes were included in the analysis. There were no gaps within the gene sequences, just shortened sequences, if needed to remove end regions with poor sequence coverage. Phylogenetic analysis of the H gene nucleotide sequences obtained from the examined samples and others obtained from the GenBank databases revealed that there are at least three different CDV lineages circulating in the US (Figs. 1 and 2).

According to the phylogenetic analysis of the H gene nucleotide sequences, 22 sequences out of 59 grouped with the America-3 (Edomex) genotype (Fig. 1, purple clade), and 8 sequences grouped with the America-4 genotype (Fig. 1, dark blue clade). Nineteen sequences clustered with a CDV strain that was previously reported in association with an outbreak in Wyoming (GenBank accession number JF283477), which was linked to a domestic dog-breeding facility in Kansas in 2010 (Fig. 1, green clade). Alignment of these “WY-like” 19 H gene nucleotide sequences revealed identity level ranges from 96.09 to 99.98% (Table 1); whereas alignment of these sequences with H gene nucleotide sequences from different available lineages showed identity level ranges from 91.12 to 95.59% (Table 2).

**Table 1:** H gene divergence/% identity between WY-like strains

	S134 GA	S99 MS	S97 MS	S95 MS	S85 MS	S26 OH	S9 Al	S56 AL	S86 MS	S23 WV	S21 WV	S22 WV	S5 TN	S55 AL	S12 TN	S18 MO	S20 TX	S8 AL	S119 KS
S134 GA																			
S99 MS	99.72																		
S97 MS	99.61	99.79																	
S95 MS	99.67	99.85	99.85																
S85 MS	99.46	99.64	99.54	99.59															
S26 OH	99.26	99.38	99.28	99.33	99.13														
S9 Al	99.36	99.54	99.44	99.49	99.28	99.54													
S56 AL	99.36	99.54	99.44	99.49	99.28	99.54	99.69												
S86 MS	99.61	99.69	99.59	99.64	99.43	99.18	99.33	99.33											
S23 WV	98.95	99.08	98.97	99.02	98.82	99.18	99.23	99.23	98.87										
S21 WV	99.05	99.18	99.08	99.13	98.92	99.28	99.33	99.33	98.97	99.9									
S22 WV	99.05	99.18	99.08	99.13	98.92	99.28	99.33	99.33	98.97	99.79	99.9								
S5 TN	98.79	98.92	98.82	98.87	98.66	99.02	99.07	99.07	98.81	99.43	99.43	99.33							
S55 AL	99.33	99.47	99.36	99.41	99.2	99.47	99.63	99.95	99.25	99.25	99.25	99.25	99.09						
S12 TN	98.84	98.97	98.87	98.92	98.72	99.08	99.13	99.13	98.81	99.08	99.08	99.08	99.07	99.15					
S18 MO	96.79	96.97	96.87	96.92	96.71	96.97	97.12	97.12	96.8	96.97	96.97	96.97	96.86	97.12	96.97				
S20 TX	97.14	97.26	97.26	97.26	96.95	97.18	97.48	97.41	97.03	97.03	97.1	97.18	97.03	97.42	96.95	98.17			
S8 AL	97.44	97.54	97.54	97.54	97.21	97.54	97.67	97.67	97.34	97.54	97.54	97.54	97.47	97.83	97.34	97.15	97.33		
S119 KS	96.91	96.97	96.97	97.1	96.84	97.1	97.22	97.22	96.84	96.97	96.97	97.1	97.1	97.23	97.22	96.59	96.09	96.84	

Evaluation of strains detected in the US that cluster with a CDV strain previously identified from a domestic dog-breeding facility in Kansas in 2010^19^. Samples represented by assigned sample (S) number and state in which the animal was located

**Table 2:** H gene divergence/% identity between WY- like sequences and representatives of CDV lineages

	A2	S134 GA	S 99 MS	S26 OH	S56 AL	S21 WV	S5 TN	WY	S18 MO	S20 TX	Eur/SA1	A4	A3	SA3	SA2	Africa	AS1	Arctic	AS2	A1
A 2																				
S134 GA	95.65																			
S99 MS	95.78	99.72																		
S26 OH2	95.78	99.26	99.38																	
S56 AL	95.89	99.34	99.49	99.49																
S21 WV	95.78	99.05	99.18	99.28	99.28															
S5 TN	95.62	98.79	98.92	99.02	99.02	99.43														
WY	96.59	97.87	98.02	98.02	98.21	97.96	97.96													
S18 MO	95.84	96.79	96.97	96.97	97.07	96.97	96.86	98.02												
S20 TX	95.75	96.99	97.11	97.03	97.26	96.96	96.88	97.76	98.02											
Eur/SA1	96.31	95.35	95.48	95.58	95.48	95.63	95.42	96.16	95.48	95.58										
A4	95.36	94.49	94.61	94.61	94.72	94.77	94.56	95.36	94.61	94.53	95.75									
A3	95.48	94.51	94.7	94.5	94.51	94.3	94.11	95.28	94.5	93.49	96.07	95.28								
SA3	95.18	94.49	94.52	94.41	94.58	94.52	94.41	94.86	94.24	94.35	95.94	94.79	95.28							
SA2	94.95	93.92	94.03	93.92	94.04	93.8	93.57	95.87	94.26	93.93	95.06	94.37	95.87	93.92						
Africa	94.52	93.56	93.7	93.59	93.75	93.59	93.42	93.87	93.86	93.12	94.79	94.19	93.52	94.24	94.03					
AS1	94.36	93.71	93.83	93.94	93.94	93.88	93.67	94.67	93.68	94.14	94.81	93.94	94.11	93.64	93.69	93.53				
Arctic	94.79	93.78	93.91	93.91	94.08	93.91	93.75	94.24	93.91	93.66	95.07	94.52	92.53	93.97	93.46	94.68	93.75			
AS2	93.89	92.99	93.12	93.12	93.12	93.01	92.85	93.81	93.12	93.15	94.09	93.32	92.34	92.82	92.08	93.42	92.7	93.64		
A1	92.62	91.61	91.73	91.83	91.84	91.78	91.57	91.7	91.58	91.4	92.75	91.66	92.14	91.83	91.16	91.89	91.06	92.32	91.01	

Evaluation of “WY-like” strains detected in the US with representatives of other CDV lineages: AF378705 (A1, America-1); Z54166 (A2, America-2); HM771716 (A3, America-3 Edomex); KJ74971(A4, America-4); JF283477^*^ (WY- 2010); AB687720 (AS1, Asia 1); AB474397 (AS2, Asia 2); FJ461723 (AFRICA); DQ226087 (Arctic); Z47761 (EU/SA1, Europe/South America 1); AM422850 (SA2, South America 2); KF835420 (SA3, South America 3). Samples from this study represented by assigned sample (S) number and state in which the dog was located

*A canine distemper strain that was previously reported in association with an outbreak in Wyoming, which was linked to a domestic dog-breeding facility in Kansas in 2010 (Fig. 1, green clade)^19^

**Fig. 3:**
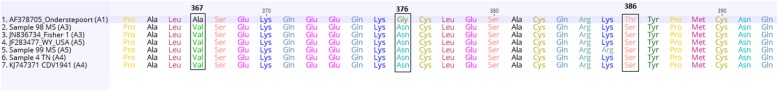
Alignment of deduced amino acid sequence located between aa residues 364 and 392 of CDV-H protein among representative CDVs selected from each lineage identified in this study with the Onderstepoort strain of the America-1 lineage

Evaluation of the deduced amino acid sequence that constitutes the major neutralizing antigenic site of morbillivirus-H protein [20] among representative sequences from each clade and the vaccine strain (America-1) revealed potential substitutions at three aa residues. The three assessed clades have valine (V), asparagine (N) and serine (S) at positions 367, 376 and 386, respectively. Whereas, the vaccine strain has alanine (A), glycine (G) and threonine (T), respectively (Fig. 3).

Additional findings included one H gene sequence that grouped with the Asia-1 genotype. Two sequences (cases 70 and 71, which were collected in 2004–2005) grouped with other H gene nucleotide sequences that were previously detected in two CDV cases in Missouri in 2004 (GenBank accession number AY964108 and AY964112). Alignment of these sequences revealed high identity (99.77–100%) to the CDV detected in Missouri’s cases in 2004 [21]. These sequences cluster with strains in the Arctic-like lineage (Fig. 1, red clade).

Four H gene nucleotide sequences (samples: 100, 101, 126 and 127) from dogs from North Dakota grouped together in an independent clade with an American dog strain that was isolated in 1992 (GenBank accession number Z47762). Alignment of these strains showed high identity (99.77 to 100%) with each other, while they only share ~ 97.5% identity with the previously described American dog strain (Table 3). There was no vaccination history available for these 4 dogs.

**Table 3:** H gene divergence/% identity between CDV sequences detected in samples collected from dogs in North Dakota and the America-1 lineage (Onderstepoort)

	S127 ND	S101 ND	S126 ND	S100 ND	Am. dog	A1
S127 ND						
S101 ND	100					
S126 ND	99.74	100				
S100 ND	99.74	99.74	99.74			
Am. dog	97.567	97.77	97.4	97.67		
A1	90.89	92.31	91.08	93.28	91.88	

Samples represented by assigned sample (S) number and state in which the dog was located. GenBank numbers: AF378705 (A1, America-1; Onderstepoort) and Z47762 (Am.dog, American dog strain from 1992)

There were 2 sequences that were obtained from a 4-month old dog from Minnesota (brain tissue and a pooled tissue sample of spleen and kidney, sequenced independently) that did not group with previously described strains. This animal was vaccinated 18 days prior to first presenting ill (respiratory signs) and did receive a booster vaccination, but the animal was euthanized because the disease progressed, with development of neurologic signs. The H gene nucleotide sequence of this strain was deposited in the GenBank (accession number MG797669).

**Fig. 4:**
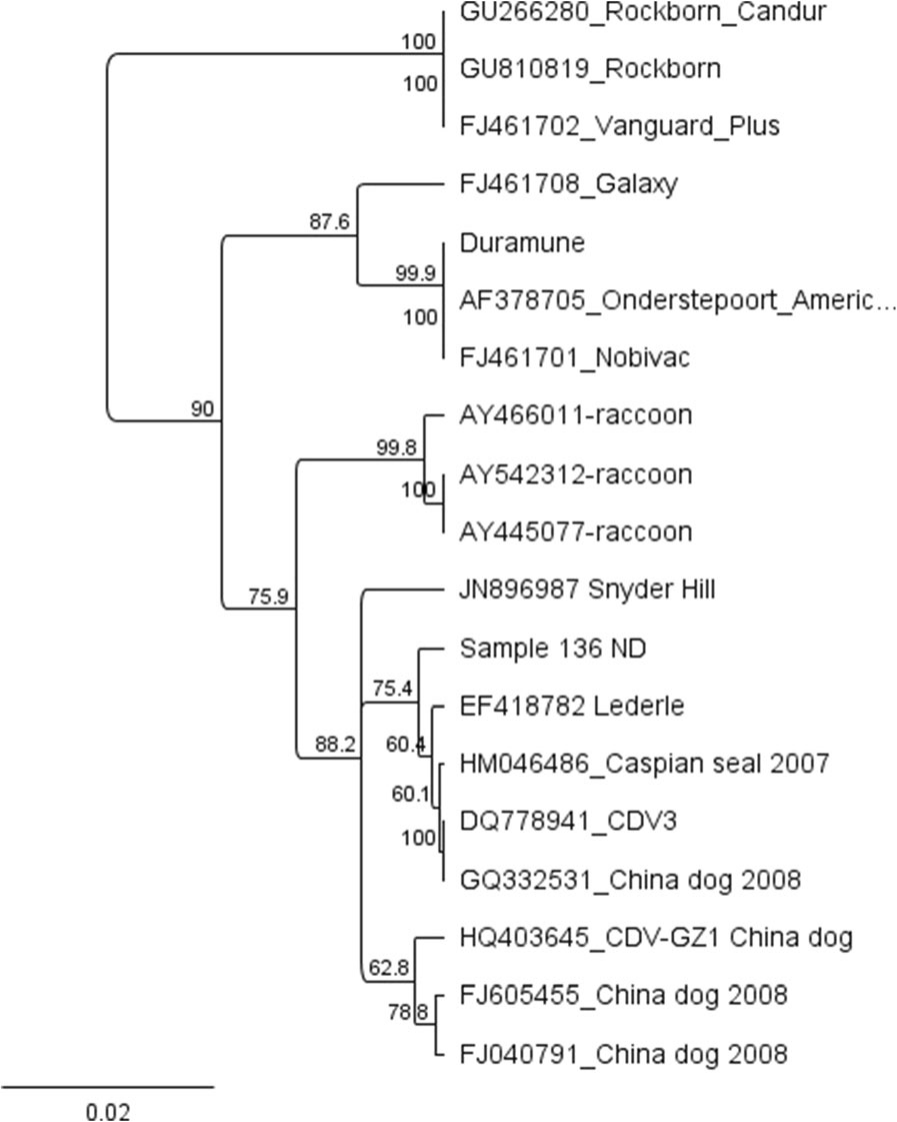
Phylogenetic analysis of the nucleotide sequences of H gene of sample 136 as compared to other CDV H gene sequences available in the GenBank. This strain was the only one detected in this study to be grouped with America-1 lineage (vaccine strains)

Interestingly, one sample grouped with the America-1 clade (Fig. 4). The animal was a Newfoundland puppy that had been vaccinated with Solo-Jec® 5 (Boehringer Ingelheim Vetmedica, Inc., St. Joseph, MO) a week prior to presenting with a fever of 103.7 °C and diarrhea. The puppy was in a litter with 3 other puppies and was housed at times in an outdoor kennel with its bitch and another bitch with a litter of 5 puppies. The location of the animals was in a rural area known to have wildlife, including coyotes, foxes, raccoons, and skunks. This puppy was the only animal that displayed any clinical signs consistent with CDV. The puppy died and on necropsy had enteritis, pneumonia, and severe thymic and splenic necrosis.

A comparison of representative CDVs selected from each lineage identified in this study was performed with the Onderstepoort strain of the America-1 lineage, which is the most common strain in the currently available vaccines. The comparison showed low identity, ranging from 91 to 93% (Table 4). Phylogenetic analyses of the nucleotide sequences obtained in this study of the F and P genes and the M-F intergenic region with sequences from the GenBank database produced similar findings to the H gene analysis. Thus, these phylogenetic trees are not included.

**Table 4:** H gene divergence / % identity among representative CDVs selected from each lineage identified in this study with the Onderstepoort strain of the America-1 lineage

	S128 ND	S100 ND	S21 WV	S14 TX	S4 TN	S70 IL	A1
S128 ND							
S100 ND	95.35						
S21 WV	95.47	96.38					
S14 TX	93.75	93.28	93.82				
S4 TN	94.24	94.57	94.82	94.87			
S70 IL	93.14	93.28	93.59	93.06	94.28		
A1	91.18	93.28	91.78	91.45	91.67	91.84	

Samples represented by assigned sample (S) number and state in which the dog was located. GenBank numbers: AF378705 (A1, America-1 Onderstepoort)

## Discussion

Canine distemper is one of the most important viral diseases in dogs worldwide. Sequence analysis of CDVs from different geographical areas has shown variation in the genome of the virus, especially in the H gene, which may result in vaccine failure [2]. Therefore, surveillance and phylogenetic analysis of wild-type circulating CDV in the US is clinically important and may reveal the need for a potent multivalent vaccine to protect against different strains of CDV [5]. The CDV H protein is involved in host cell binding and shows more variability among CDV isolates. Thus the H gene is a suitable target for molecular epidemiological studies. Furthermore, the majority of neutralizing epitopes are found on the H protein, making this gene also important for evaluation of changes over time that may result in antigenic differences among strains. There are many more complete H gene sequences than any other CDV gene sequences available in public databases for phylogenetic comparisons. Analysis of the H gene sequences revealed that there are at least three main lineages circulating in the US. None of these lineages show a close genetic relationship to the most commonly used CDV vaccine strain, Onderstepoort (Fig. 1 and Table 4). Most CDV vaccines have been manufactured using America-1 lineage strains (Onderstepoort, Snyder Hill, Lederle), which were isolated between the 1940s and 1960s [22, 23]. The genetic variation observed among the circulating CDVs and America-1 strains may be associated with antigenic variation that is sufficient to prevent currently available vaccines from providing complete immune protection [5]. To assess this hypotheses, we should evaluate the major deduced antigenic epitopes of the examined CDV-H protein. However, due to lack of information about the amino acid residues or regions that constitute these epitopes, we extrapolated data to assess what is considered the most immunodominant epitope of morbillivirus [4]. This major neutralizing antigenic site of morbillivirus should theoretically be located between aa residues 364 and 392 of CDV-H protein [4, 20]. It should be noted that CDV and measles virus (MV) H protein numbering vary by 4 aa (i.e the corresponding region in MV is located between aa 368-396) [24]. This epitope in MV is known as hemagglutinating and noose epitope (HNE) because three cysteines in the epitope form a surface exposed loop. The disulfide constrained surface exposed loop is often described as the noose motif [25]. Analysis of this region among representative CDV-H protein from each clade and the vaccine strain revealed that there are substitutions at three aa between the circulating strains and the vaccine strain. HEN has been implicated to play a role in the function or the conformational stability of the MV-H protein [26]. It has been demonstrated that monoclonal antibodies that recognize measles HNE epitope neutralize MV infection by blocking the virus binding to its receptor [26]. Therefore, we suggest that the predicted substitutions observed in this epitope may interfere with the ability of the vaccine to provide adequate protection against infection with these strains [5].

Vaccine failure was suspected in one case in this study (case 133) in a 2-year-old dog that died and was diagnosed with CDV on necropsy. This animal was infected with the America-3 strain. The dog had been rescued and vaccinated (Nobivac Canine-1 DAPPV, Merck Animal Health, Madison, NJ) three months prior to a CDV outbreak that occurred in the shelter in which the animal was being housed. When this America-3 lineage was originally described in Mexico, it was detected from two dogs, approximately 1 year of age that had also been vaccinated 2–3 months prior to developing CD clinical signs [16].

Movement of dogs from country to country or state to state is common and can spread CDV strains into new areas. This study has confirmed the spread of the America-4 strain from the Southeast [14] to the Midwest region of the US. Additionally, the America-3 strain (Edomex), which as mentioned, is in Mexico [16], but it is also widely distributed throughout the US (Fig. 2). We discovered an Asia-1 strain in a sample from a dog located in New Jersey. According to the history in this particular case, the dog was brought to the US from South Korea and therefore was most likely incubating the virus during transport to the US.

In this study, we have also obtained additional evidence to prove that the Arctic-like lineage was circulating in the US in 2004–2005. Two sequences of CDV that were detected in samples collected in 2004 and 2005 from dogs in Illinois clustered with other CDV isolates that were previously sequenced from Missouri in 2004 into an independent clade with Arctic-like lineage strains. The dogs in the Missouri study had never left that state. When the sequences of the Missouri CDV strains were published in 2005, it was the first time to document Arctic-like strains in the US [21]. The previous publication failed to link the Missouri strains to the Arctic-like lineage because the lineage had not been well defined at that point. Isolation of an Arctic-like strain in 2007 from a 10-week-old, CDV-vaccinated Weimaraner dog from Missouri has also been reported in the literature [17], suggesting the strain circulated in the US for several years. We did not detect Arctic-like strains from current cases of CD in the US.

In 19 samples out of 59 tested, the nucleotide sequences of the CDV detected grouped with CDV isolated [27] from a canine breeding facility in Wyoming in 2010 in one clade, with limited heterogeneity between strains over the period from detection until now. While most closely related to America-2 strains, these sequences cluster together to form an independent clade and appear to represent a distinct lineage that has been circulating in the US since at least 2010. Therefore, for ease of reference, we propose naming this lineage “America-5”.

A different strain appears to be circulating in ND, which was found in 4 dogs within the last year. Alignment of the H gene nucleotide sequences of these 4 CDV determined high level of identity (99.7–100%) with each other and ~ 97.5% identity to a previously described American dog strain [2]. The difference between these two strains and the previously described strain may reflect changes that occurred to the American dog strain over the time. More surveillance is needed to determine if this strain will remain in the population and how widespread it is.

Unexpectedly, we detected an America-1 strain in a puppy (Fig. 4). We suspected this may have been a vaccine related case based on the history but discovered with sequencing that the strain was unique from the vaccine strain the dog had been given. America-1 strains have not been documented in recent history in dogs in the US but were found in samples collected from raccoons in 1998 in the US [28] and from a seal in the Caspian Sea region in 2007 (GenBank accession number HM046486). There are however, America-1 strains in GenBank obtained from dogs in China from samples collected in 2008 (Fig. 4). The most likely explanation for the particular animal in this study is that it was exposed to the America-1 strain from wildlife. While the vaccine should have provided protection, given that it is also of the America-1 clade, the animal was likely already incubating the virus when vaccinated or was potentially a non-responder. However, given this finding, it suggests the America-1 clade is still circulating in wildlife in the US and the current vaccines are effectively keeping these strains out of the domestic dog population.

Though the designed primers were not able to amplify the complete CDV genome, the partially amplified CDV genome sequences of various CDV strains showed the ability of the developed targeted NGS assay to amplify CDV strains with real time Ct values as high as 33. Full genome sequences of some lineages were not available in GenBank and based on discovered sequence differences to our designed primers, additional primers should be added to the design to achieve full genome sequencing for multiple strain types. Missing regions could also be filled in using conventional PCR and Sanger sequencing, but this is beyond the scope of this work. This method of targeted whole genome sequencing was employed to increase the sensitivity of detection of the virus among a large amount of host nucleic acids in tissues. Evaluation of whole genomes provides more data than analysis of the H gene alone and may uncover recombination events that would not be detected otherwise, though none were detected in these sequences. While the method used for this study did not providing complete genomes, the method was successful for amplifying CDV genes in a variety of sample types, including formalin-fixed paraffin embedded tissues.

## Conclusion

In summary, movement of dogs from country to country or state to state is common and can spread CDV strains into new areas. There are 3 main lineages of CDV currently circulating in the US, designated America-3, America-4, and America-5. These lineages differ from the historically identified lineages in the US, including America-1, which contains the majority of the vaccine strains. Genetic differences can result in significant changes to the neutralizing epitopes that consequently may lead to vaccine failure. Continuous surveillance is required for monitoring circulating CDV strains to prevent potential vaccine breakthrough events. Further study is required to determine whether these genetic variations represent significant differences in antigenicity, particularly between vaccine strains and these wild-type strains circulating in the USAdditional file 1.

## Additional file

Additional file 1: CDV primer sequences. (XLS 120 kb)

## Abbreviations

CD: Canine distemper
CDV: Canine distemper virus
F: Fusion proteins
H: Hemagglutinin proteins
NGS: Next-generation sequencing (NGS)
RT-PCR: Reverse transcription polymerase chain reaction
